# *Antrodia cinnamomea* extract exhibits anti-melanogenic and anti-photoaging effects: potential for cosmetic and dermatological applications

**DOI:** 10.1038/s41598-026-54214-8

**Published:** 2026-05-28

**Authors:** I-Hsuan Huang, Tse-Kai Fu, Yu-Ting Chou

**Affiliations:** 1 Institute of Biotechnology, National Tsing Hua University, No. 101, Section 2, Kuang-Fu Road, Hsinchu, 30013 Taiwan; 2 Simpson Biotech Co., Ltd., Taoyuan, Taiwan

**Keywords:** Cell signalling, Ageing, Skin diseases

## Abstract

Skin pigmentation and photoaging are major dermatological concerns, underscoring the need for safe and effective topical agents. *Antrodia cinnamomea*, a medicinal mushroom native to Taiwan, is well known for its hepatoprotective, anti-cancer, and metabolic benefits; however, its anti-melanogenic and anti-photoaging potential remains insufficiently characterized. In this study, we demonstrate that *A. cinnamomea* ethanol extract (AC-EtOH) suppresses melanin production and tyrosinase activity in α-MSH–stimulated B16F10 melanocytes by downregulating the mRNA expression of tyrosinase, TRP-1, TRP-2, and microphthalmia-associated transcription factor (MITF). AC-EtOH also inhibits α-MSH–activated PKA/cAMP/CREB signaling, further contributing to melanogenesis suppression. In skin fibroblasts, AC-EtOH protects against UV-induced photoaging by reducing the accumulation of reactive oxygen species (ROS) and attenuating the expression of matrix metalloproteinases (MMP1 and MMP3) and pro-inflammatory cytokines (IL-1β and IL-6). To improve its suitability for cosmetic formulations, polyethylene glycol (PG)—a widely used solvent—was employed to generate an *A. cinnamomea* extract–PG mixture (AC-PG). AC-PG retained the biological efficacy of AC-EtOH, reducing melanin production and tyrosinase activity in α-MSH–induced cells, while exhibiting low sensitization and irritation potential, as per OECD Test Guidelines 439, 492, and 442E. Collectively, these findings identify AC-EtOH as a promising natural ingredient with potent anti-melanogenic and anti-photoaging activities, supporting its potential application in cosmetic and dermatological products for skin whitening, anti-wrinkle, and anti-photoaging purposes.

**Supplementary Information:**

The online version contains supplementary material available at 10.1038/s41598-026-54214-8.

## Introduction

Melanin is the primary pigment responsible for skin color and serves a protective role against UV radiation. However, excessive melanin production, abnormal accumulation, or uneven distribution can lead to hyperpigmentation disorders such as age spots, freckles, melasma, and speckles^1^. Melanogenesis is a complex, multi-step process involving both enzymatic and chemical reactions^2^. Tyrosinase, the rate-limiting enzyme in melanin biosynthesis, catalyzes the oxidation of tyrosine to L-DOPA and subsequently to L-dopaquinone^3^. Tyrosinase-related protein-1 (TRP-1) and tyrosinase-related protein-2 (TRP-2) contribute specifically to eumelanin synthesis^4,5^. TRP-2 converts dopachrome to 5,6-dihydroxyindole-2-carboxylic acid (DHICA), which TRP-1 further oxidizes into a carboxylated indole-quinone, forming eumelanin^5,6^. Microphthalmia-associated transcription factor (MITF) is a key regulator of melanogenesis, controlling the expression of tyrosinase, TRP-1, and TRP-2 in melanocytes^7,8^. MITF activity is modulated by the PKA/cAMP/CREB pathway^5,9,10^, where α-melanocyte-stimulating hormone (α-MSH) binds to the melanocortin-1 receptor (MC1R), increasing intracellular cAMP levels and activating PKA^11,12^. PKA phosphorylates cAMP-response element-binding protein (CREB), which enhances MITF transcription. Since tyrosinase is crucial for melanin biosynthesis, its regulation is a key strategy for anti-melanogenesis, making it a primary target in the development of skin-lightening agents.

UV radiation is a significant extrinsic factor that contributes to skin aging, commonly referred to as photoaging. Photoaging manifests as deep wrinkles, skin atrophy, reduced elasticity, hyperpigmentation, and inflammation^13,14^. UV exposure generates reactive oxygen species (ROS) in the dermal layer, initiating signaling cascades that accelerate skin damage^15^. Specifically, UV radiation activates activator protein-1 (AP-1) via the mitogen-activated protein kinase (MAPK) pathway, leading to the upregulation of matrix metalloproteinases (MMPs), such as MMP1 and MMP3, which degrade collagen fibrils and contribute to wrinkle formation^16–18^. Additionally, UV-induced ROS activates NF-κB signaling, increasing MMP expression and promoting the secretion of pro-inflammatory cytokines such as TNF-α, IL-1β, and IL-6, exacerbating skin inflammation^18,19^. Therefore, eliminating UV-induced ROS is essential for preventing photoaging.

The demand for skin-lightening and anti-aging products has driven the development of cosmetic formulations targeting melanogenesis and photoaging^20^. While synthetic agents such as hydroquinone, kojic acid, and retinoids are widely used, they are associated with adverse effects, including mutagenicity, skin irritation, dryness, and inflammation^20,21^. This has led to a growing interest in natural compounds as safer alternatives. *Antrodia cinnamomea*, a medicinal mushroom endemic to Taiwan, has gained recognition as the “forest ruby” due to its potent therapeutic properties^22^. It has demonstrated preventive and therapeutic effects against hepatitis, cancer, hyperlipidemia, and other diseases^23^. Extracts of *A. cinnamomea* and its bioactive compounds exhibit potent antioxidant and anti-inflammatory activities in the liver, lungs, and skin^24–26^. However, its potential in anti-melanogenesis and anti-photoaging remains incompletely understood. In this study, we investigated the effects of *A. cinnamomea* ethanolic extract (AC-EtOH) on melanogenesis inhibition and UV-induced skin aging, evaluating its feasibility for cosmetic and skincare applications.

## Materials and methods

### Cell culture

B16F10, WS1, and THP-1 cell lines were obtained from the Bioresource Collection and Research Center (BCRC, Taiwan). B16F10 and WS1 cells were maintained in Dulbecco’s Modified Eagle Medium (DMEM) and Minimum Essential Medium α (MEM-α), respectively, whereas THP-1 cells were cultured in RPMI-1640 medium. All media were supplemented with 10% fetal bovine serum (FBS; Hyclone), 100 µg/mL streptomycin, and 100 U/mL penicillin. Cells were incubated at 37 °C in a humidified atmosphere containing 5% CO₂.

### ***A. cinnamomea*** ethanol extract (AC-EtOH) preparation

*A. cinnamomea* mycelium was produced by Simpson Biotech Co., Ltd. using submerged liquid fermentation as described by Stamford et al. (“Protein Enrichment of Cashew Wastes for Animal Feeds,” UNU Food Science). The mycelium was mixed with ethanol at a 1:5 (w/w) ratio, heated to 60–65 °C with stirring, and then cooled to 40 °C. The liquid fraction was collected using a separator and concentrated under reduced pressure to obtain the ethanol extract (AC-EtOH). AC-EtOH was dissolved in DMSO for all experiments.

### Cell viability assay

Cell viability was determined using the Cell Counting Kit-8 (CCK-8; Dojindo, Japan). B16F10 (1 × 10³ cells/well) and WS1 (2.5 × 10³ cells/well) were seeded into 96-well plates and treated with various concentrations of AC-EtOH for the indicated durations. Cells were then incubated with 10% CCK-8 reagent for 2 h at 37 °C. Absorbance at 450 nm was measured using a microplate reader.

### Melanin content assay

B16F10 cells (5 × 10⁴/well) were seeded in 6-well plates and treated with AC-EtOH in the presence of 100 nM α-MSH (Sigma-Aldrich, USA) for 48 h. Intracellular melanin: Cells were harvested, lysed in 1 N NaOH containing 10% DMSO, and heated at 70 °C for 1 h. Absorbance was measured at 405 nm.

Extracellular melanin: Culture supernatants were collected, and melanin levels were quantified at 490 nm.

### Tyrosinase activity assay

Following AC-EtOH treatment, B16F10 cells were lysed in 50 mM sodium phosphate buffer (pH 6.8) containing 1% Triton X-100 and protease inhibitors. Lysates were centrifuged at 13,000 rpm for 15 min at 4 °C. Supernatants (80 µL) were mixed with 20 µL of 2 mg/mL L-DOPA (Sigma-Aldrich) and incubated at 37 °C for 30 min. Tyrosinase activity was measured at 490 nm and normalized to protein concentration (Bradford assay). In vitro tyrosinase activity was assessed using mushroom tyrosinase. Briefly, 80 μL of mushroom tyrosinase solution (25 U) was incubated with various concentrations of AC-EtOH in sodiun phosphate buffer (pH 6.8) at 37 °C for 5 min. Subsequently, 20 μL of 2 mg/mL L-DOPA was added to initiate the reaction, followed by incubation at 37 °C for 10 min. Tyrosinase activity was measured at 490 nm.  

### cAMP assay

Intracellular cAMP levels were determined using the Cyclic AMP XP^®^ Assay Kit (Cell Signaling, USA). B16F10 cells (2 × 10⁴/well) were seeded in 96-well plates, pre-incubated overnight, and treated with AC-EtOH and 100 nM α-MSH for 15 min. Cells were then lysed on ice for 10 min, and supernatants were subjected to ELISA. cAMP concentrations were calculated from standard curves.

### UV irradiation

WS1 cells were seeded in 30-mm dishes, washed with PBS, and covered with 0.75 mL PBS. Cells were exposed to 40 mJ/cm² UVB using UVB lamps (Nomo, China). After irradiation, cells were cultured in complete medium containing the indicated concentrations of AC-EtOH for 24 h.

### Intracellular ROS assay

ROS were detected using DCF-DA (Sigma-Aldrich). After UVB exposure and AC-EtOH treatment, cells were incubated with 40 µM DCF-DA for 30 min at 37 °C, washed with PBS, and imaged using an inverted fluorescence microscope (Zeiss). For quantification, cells were lysed in RIPA buffer, and fluorescence intensity was measured at an excitation/emission wavelength of 485/530 nm using a VICTOR NIVO plate reader. Fluorescence values were normalized to protein concentration.

### NF-κB reporter luciferase assay

NF-κB transcriptional activity was measured using the Dual-Luciferase^®^ Reporter Assay System (Promega). WS1 cells were transfected with the pGreenFire 2.0 NF-κB reporter plasmid (System Biosciences). After UVB exposure and AC-EtOH treatment, luciferase activities were determined according to the manufacturer’s instructions and normalized to total protein.

### qPCR analysis

Total RNA was extracted using TRIzol reagent. One microgram of RNA was reverse-transcribed using the PrimeScript RT Reagent Kit (TaKaRa). qPCR was performed using TaqMan probes or Universal Probe Library probes (Roche Applied Science) on a StepOne System (Applied Biosystems). Gene expression was normalized to 18 S or GAPDH. All experiments were conducted in triplicate. Primer and probe sequences are listed in Table S1.

### Western blot analysis

Cells were lysed in RIPA buffer supplemented with protease and phosphatase inhibitors. Lysates were sonicated and centrifuged at 12,500×*g* for 30 min at 4 °C, and the resulting supernatants were collected. Protein concentrations were determined using the Bradford assay. Equal amounts of protein (30 µg) were resolved on 10% SDS–PAGE gels and transferred onto nitrocellulose membranes. Protein molecular weights were determined using the ExcelBand 3-Color Regular Range Protein Marker [PM2500] (SMOBIO; Cat# PM2500). Membranes were blocked with 5% non-fat milk for 1 h at room temperature (RT) and incubated overnight at 4 °C with the appropriate primary antibodies. After washing with TBST, membranes were incubated with HRP-conjugated secondary antibody (GeneTex, GTX213110-01; 1:10,000) for 2 h at RT. Protein signals were detected using enhanced chemiluminescence reagents and visualized with an LS4000 Imaging System. The MMP3 primary antibody was purchased from Cell Signaling Technology (USA), and all other primary antibodies were obtained from GeneTex (Taiwan). Antibodies were used at the following dilutions: CREB (GTX112846, 1:1000), phospho-CREB (GTX1310379, 1:500), tyrosinase (GTX16389, 1:1000), MMP1 (GTX1005334, 1:2000), MMP3 (D7F5B, 1:1000), TIMP1 (GTX108254, 1:1000), and GAPDH (GTX100118, 1:10,000).

### Cytokine quantitation

Secreted IL-6 and IL-1β levels were measured using commercial ELISA kits (Arigo Biolaboratories, Taiwan). WS1 cells (1 × 10⁵/well) were seeded in 24-well plates, exposed to UVB and AC-EtOH, and supernatants were collected. Cytokine concentrations were determined from standard curves.

### Immunofluorescence analysis

WS1 cells (5 × 10⁴/well) were seeded on collagen-coated coverslips. Following UVB irradiation and AC-EtOH treatment, cells were fixed with 4% formaldehyde for 15 min, washed with PBS, and blocked with PBS containing 5% BSA and 0.3% Triton X-100 for 1 h. Cells were incubated with anti-NF-κB p65 antibody (Cell Signaling Technology) overnight at 4 °C, followed by DyLight 488-conjugated anti-rabbit IgG (GeneTex) for 2 h. Nuclei were stained with DAPI (1 µg/mL). Images were acquired using a Zeiss Apotome microscope. Nuclear translocation was quantified as the ratio of nuclear to cytoplasmic fluorescence intensity using ImageJ.

### Human cell line activation test (h-CLAT)

Skin sensitization potential was assessed according to the OECD Test Guideline (TG) 442E^27^. The skin sensitization potential was assessed according to the OECD Test Guideline (TG) 442E. THP-1 cells (1 × 10⁶/well) were treated with AC-PG for 24 h, collected, and washed with PBS containing 1% BSA. Cells were stained on ice for 25 min with FITC-conjugated anti-CD86, anti-CD54, or IgG1 isotype control antibodies. After washing, cells were resuspended in PBS containing 7-AAD and analyzed using a CytoFLEX flow cytometer (Beckman Coulter). Relative fluorescence intensity (RFI) was calculated as: RFI(%)= (MFI_test_ − MFI_test isotype)_/(MFI_solvent_ − MFI_solvent isotype_) ×100. Sensitization was considered positive when CD86 RFI ≥ 150% or CD54 RFI ≥ 200%, with cell viability ≥ 50%. FITC-conjugated antibodies were obtained from GeneTex.

### In vitro skin and eye irritation test

Skin irritation was assessed using the reconstructed human epidermis (RhE)/EpiDerm™ model (OECD TG 439), and eye irritation using reconstructed human cornea-like epithelium (RhCE)/EpiOcular™ (OECD TG 492)^28^. Briefly, EpiDerm™ and EpiOcular™ tissues were exposed to AC-PG for 1 h and 30 min, respectively, at 37 °C in a humidified 5% CO₂ atmosphere. Following exposure, the tissues were thoroughly rinsed with PBS and subsequently incubated in fresh medium for 42 h (2 h for EpiOcular™). Cell viability was determined using the MTT assay, in which tissues were incubated with medium containing 1 mg/mL MTT for 3 h, and absorbance was measured at 570 nm. According to these guidelines, RhE and RhCE tissue viabilities below 50% and 60%, respectively, indicate irritation.

### Statistical analysis

Statistical analyses were performed using GraphPad Prism 8.0.1 (GraphPad Software). Data are presented as mean ± standard deviation (SD). Statistical significance was determined using Student’s t-test, with *p* < 0.05 considered significant.

## Results

### *A. cinnamomea* extract suppresses α-MSH-induced melanogenesis

**Fig. 1:**
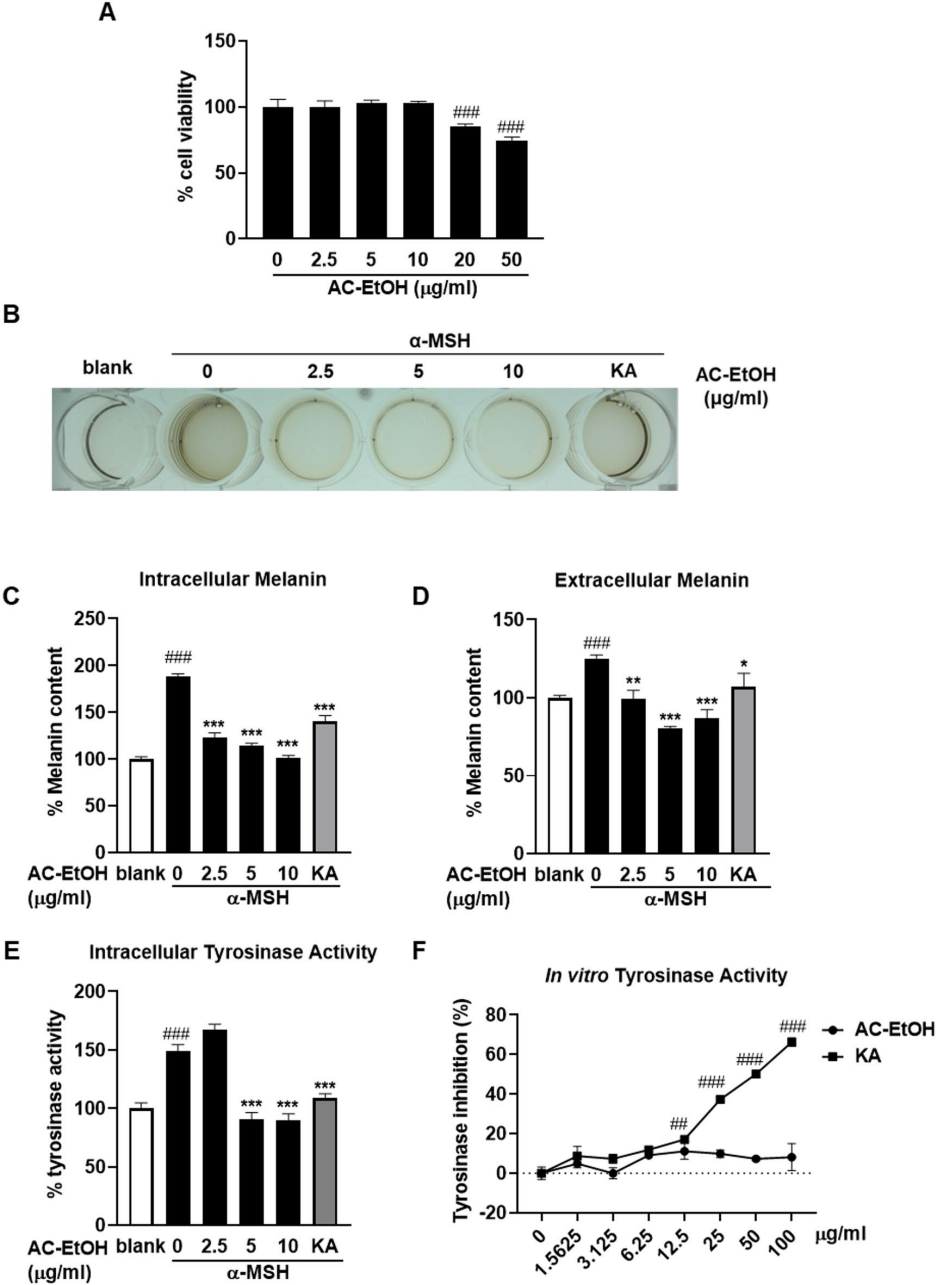
*A. cinnamomea* extract reduces melanin production and tyrosinase activity in the α-MSH-induced B16F10 melanocyte model. (**A**) CCK-8 assay measuring cell viability of B16F10 cells treated with the indicated concentrations of AC-EtOH for 48 h. ###*p* < 0.001 compared to the vehicle control group. (**B**) Representative image showing melanin content in α-MSH-induced B16F10 cells treated with the indicated concentrations of AC-EtOH for 48 h. Kojic acid (KA; 100 µg/mL) was used as a positive control. (**C**, **D**) Intracellular and extracellular melanin content measured in α-MSH-induced B16F10 cells treated with the indicated concentrations of AC-EtOH for 48 h. ###*p* < 0.001 compared to the vehicle control group; **p < 0.01 and ***p < 0.001 compared to the α-MSH control group. (**E**) Tyrosinase activity assay in α-MSH-induced B16F10 cells treated with the indicated concentrations of AC-EtOH for 48 h. ###*p* < 0.001 compared to the vehicle control group; ***p < 0.001 compared to the α-MSH control group. (**F**) Mushroom tyrosinase inhibition assay in the indicated concentrations of AC-EtOH or Kojic acid. Kojic acid served as a positive control for inhibiting mushroom tyrosinase activity. ##*p* < 0.01 and ###*p* < 0.001 compared to the vehicle control group.

To assess the cytotoxic effects of AC-EtOH on B16F10 cells, we performed a CCK-8 assay to evaluate cell viability. As shown in Fig. 1A, AC-EtOH at concentrations below 10 µg/mL did not exhibit cytotoxicity. Based on this, subsequent experiments were conducted using concentrations below 10 µg/mL to avoid adverse effects on cell viability. Melanogenesis in B16F10 cells is induced by α-MSH^29^. To investigate the inhibitory effects of AC-EtOH on melanin synthesis, B16F10 cells were co-treated with α-MSH and AC-EtOH, followed by quantification of extracellular and intracellular melanin content. Kojic acid (KA; 100 µg/mL) was used as a positive control. As shown in Fig. 1B–D, AC-EtOH significantly reduced extracellular and intracellular melanin content in a dose-dependent manner. Furthermore, AC-EtOH suppressed intracellular tyrosinase activity in α-MSH-induced B16F10 cells (Fig. 1E). However, AC-EtOH did not directly inhibit tyrosinase activity in a cell-free assay (Fig. 1F), suggesting that its anti-melanogenic effects are not due to direct tyrosinase inhibition.

### *A. cinnamomea* extract deregulates melanogenesis-related gene expression via the PKA/cAMP/CREB pathway

**Fig. 2:**
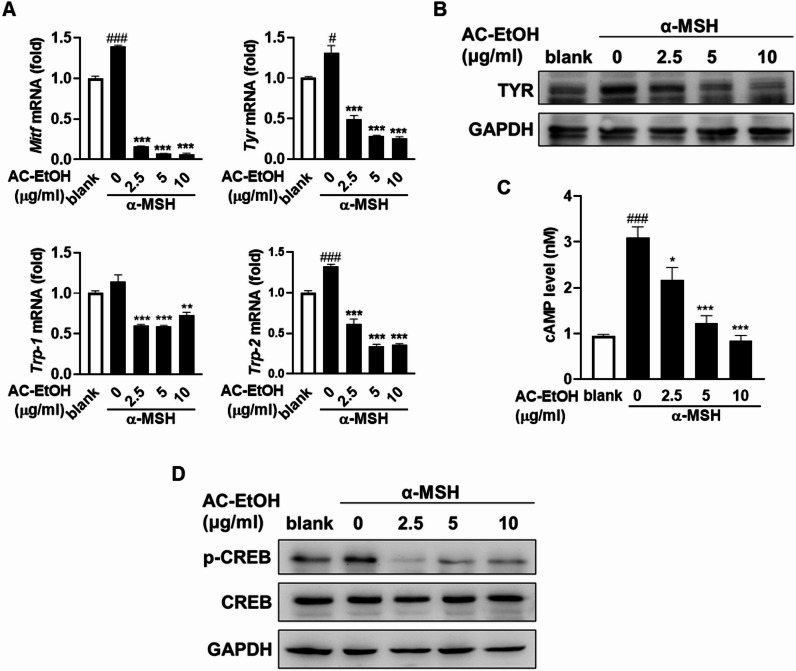
*A. cinnamomea* extract inhibits melanogenesis-related genes. (**A**) qRT-PCR analysis of *Mitf* (upper left), *Tyr* (upper right), *Trp-1* (lower left), and *Trp-2* (lower right) expression in α-MSH-induced B16F10 cells treated with the indicated concentrations of AC-EtOH for 48 h. #*p* < 0.05 and ###*p* < 0.001 compared to the vehicle control group; **p < 0.01 and ***p < 0.001 compared to the α-MSH control group. (**B**) Western blot analysis of tyrosinase (TYR) protein levels in α-MSH–induced B16F10 cells treated with AC-EtOH for 48 h. (**C**) Intracellular cAMP levels measured by ELISA in α-MSH–induced B16F10 cells treated with the indicated concentrations of AC-EtOH for 15 min. ###*p* < 0.001 compared to the vehicle control group; *p < 0.05 and ***p < 0.001 compared to the α-MSH control group. (**D**) Western blot analysis of phosphorylated CREB and total CREB levels in α-MSH-induced B16F10 cells treated with the indicated concentrations of AC-EtOH for 48 h.

Tyrosinase, TRP-1, and TRP-2 are key enzymes involved in melanogenesis^5,6^, while MITF functions as an upstream transcription factor regulating their expression^7^. To further elucidate the anti-melanogenic mechanism of AC-EtOH, we analyzed the expression of melanogenic genes in α-MSH-induced B16F10 cells. As shown in Fig. 2A, AC-EtOH treatment resulted in a significant, dose-dependent reduction in the mRNA expression levels of *Mitf*, *Tyr*, *Trp-1*, and *Trp-2*, with *Mitf* being the most affected. Consistently, AC-EtOH substantially reduced α-MSH–induced tyrosinase (TYR) protein levels (Fig. 2B). Because α-MSH triggers melanogenesis primarily through activation of the PKA/cAMP/CREB pathway^5,9,10^, we next assessed whether AC-EtOH modulates this signaling axis. The cAMP assay revealed that AC-EtOH significantly attenuated the α-MSH–induced elevation of intracellular cAMP levels (Fig. 2C). In agreement with this result, Western blot analysis demonstrated that while AC-EtOH did not affect total CREB protein levels, it significantly reduced α-MSH-induced CREB phosphorylation (Fig. 2D). These findings suggest that AC-EtOH suppresses melanogenesis by inactivating the PKA/cAMP/CREB signaling pathway.

### *A. cinnamomea* extract mitigates UV-induced cytotoxicity and reduces ROS accumulation in skin fibroblast cells

**Fig. 3:**
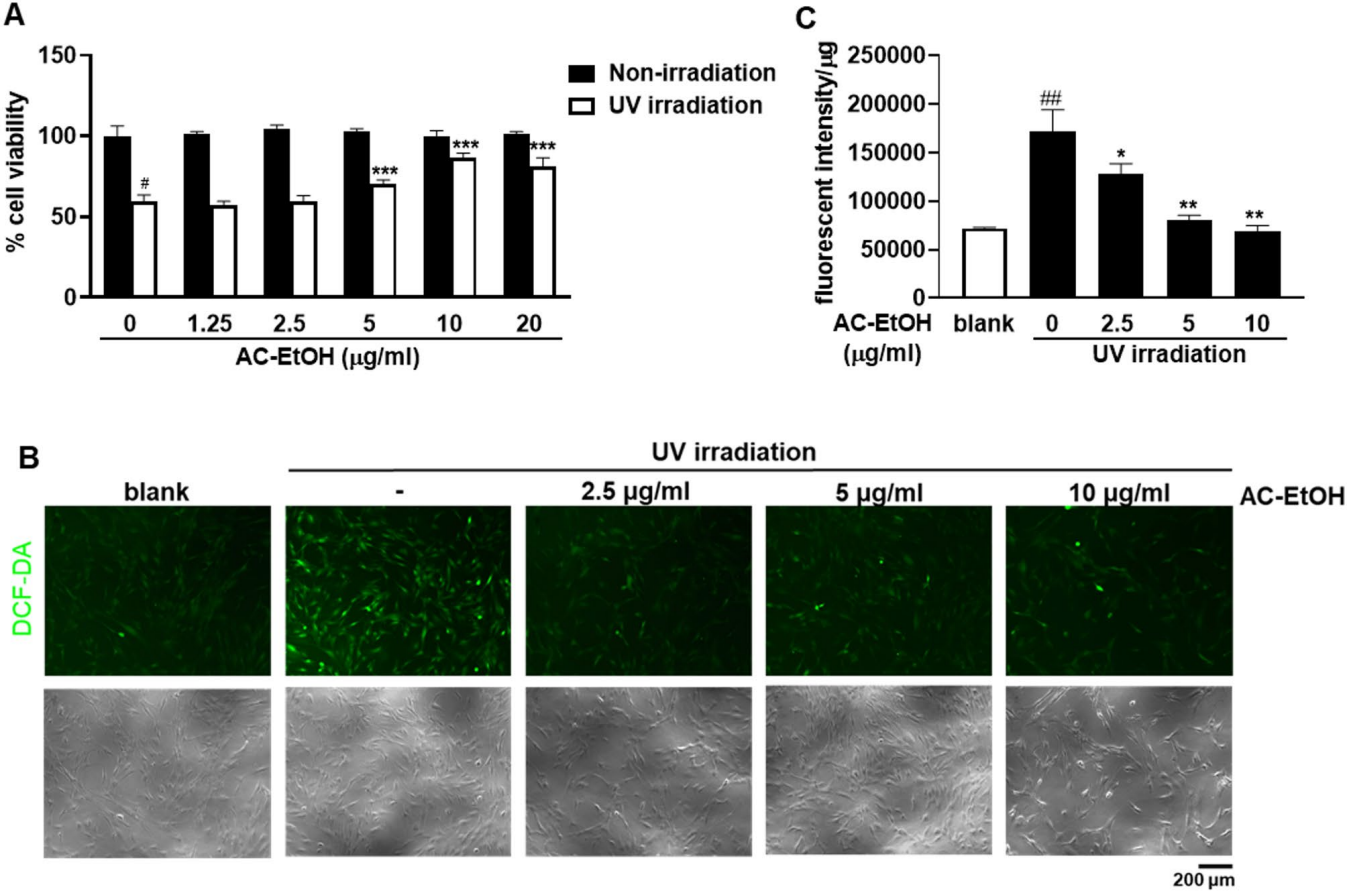
*A. cinnamomea* extract reduces UV-induced cell death and ROS in the skin fibroblast WS1 model. (**A**) CCK-8 assay measuring cell viability of WS1 cells treated with or without 40 mJ/cm² of UVB, followed by treatment with the indicated concentration of AC-EtOH for 24 h. #*p* < 0.05 compared to the non-irradiated control group; ***p < 0.001 compared to the UV-irradiated control group. (**B**, **C**) Representative image (**B**) of DCF-DA staining of WS1 cells treated with or without 40 mJ/cm² of UVB, followed by treatment with the indicated concentration of AC-EtOH for 24 h. Quantification (**C**) of intracellular ROS levels measured by DCF-DA fluorescent intensity. Scale bars, 200 μm. ##*p* < 0.01 compared to the non-irradiated control group; *p < 0.05 and **p < 0.01 compared to the UV-irradiated control group.

Inflammatory responses often accompany UV-induced cellular damage. Consistent with this, *IL-1β* mRNA expression was markedly elevated in WS1 cells exposed to 40 mJ/cm² UVB, a dose equivalent to approximately 20 min of mid-summer sunlight (Figure S1A). To assess whether AC-EtOH protects WS1 cells from UV-induced cytotoxicity, we first evaluated its effects on cell viability using the CCK-8 assay. As shown in Fig. 3A, AC-EtOH did not exhibit cytotoxicity at concentrations up to 20 µg/mL. UV irradiation resulted in 40.53% cell death in WS1 cells, whereas treatment with 5, 10, and 20 µg/mL of AC-EtOH increased cell viability by 10.97%, 29.79%, and 21.77%, respectively. UV exposure is known to generate reactive oxygen species (ROS), which contribute to cellular damage, including apoptosis, cell cycle arrest, and senescence^30^. To determine whether AC-EtOH reduces UV-induced oxidative stress, we measured ROS levels using DCF-DA staining. As illustrated in Fig. 3B, C, AC-EtOH significantly decreased ROS accumulation in UV-irradiated WS1 cells in a dose-dependent manner. N-acetyl-cysteine (NAC; 1 mM), a known ROS scavenger, served as a positive control. Consistently, Figure S1B demonstrates that both AC-EtOH and NAC diminished UV-induced ROS generation. Together, these findings suggest that AC-EtOH functions as an effective ROS scavenger, potentially enhancing cellular resilience against UV-induced oxidative stress.

### *A. cinnamomea* extract downregulates genes involved in UV-induced ECM degradation and inflammation in skin fibroblast cells

**Fig. 4:**
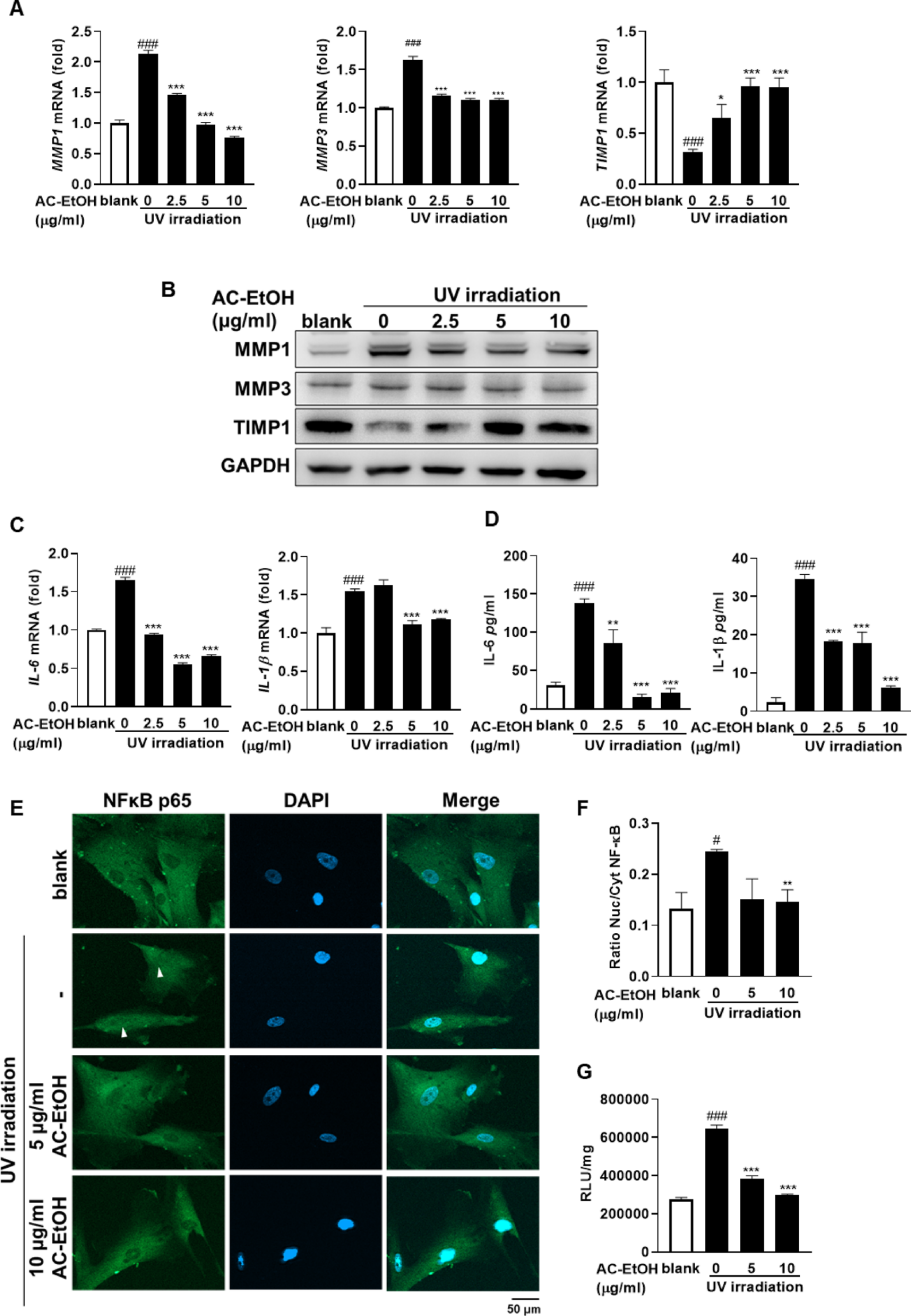
*A. cinnamomea* extract inhibits UV-induced ECM remodeling and inflammatory gene expression. (**A**, **B**) qRT-PCR analysis (A) of *MMP1* (left), *MMP3* (middle), and *TIMP1* (right) mRNA expression, and Western blot analysis (**B**) of MMP1, MMP3, and TIMP1 protein levels in UV-irradiated WS1 cells treated with the indicated concentrations of AC-EtOH for 24 h. ###*p* < 0.001 compared to the non-irradiated control group; *p < 0.05 and ***p < 0.001 compared to the UV-irradiated control group. (**C**–**D**) qRT-PCR analysis (**C**) of *IL-6* (left) and *IL-1β* (right) mRNA expression, and ELISA (**D**) quantifying IL-6 (left) and IL-1β (right) secretion in UV-irradiated WS1 cells treated with AC-EtOH for 24 h. ###*p* < 0.001 compared to the non-irradiated control group; **p < 0.01 and ***p < 0.001 compared to the UV-irradiated control group. (E-F) Representative immunofluorescence images (**E**) showing NF-κB p65 nuclear translocation in UV-irradiated WS1 cells treated with AC-EtOH for 12 h, and quantitative analysis (**F**) of nuclear translocation. Scale bars, 50 μm. #*p* < 0.05 compared to the non-irradiated control group; **p < 0.01 and compared to the UV-irradiated control group. (**G**) Luciferase reporter assay measuring NF-κB activity in WS1 cells transfected with the NF-κB reporter plasmid, exposed to UV irradiation, and then treated with the indicated concentration of AC-EtOH for 24 h. ###*p* < 0.001 compared to the non-irradiated control group; *p < 0.05 and ***p < 0.001 compared to the UV-irradiated control group.

Excessive collagen degradation weakens connective tissue integrity, leading to wrinkle formation^18^. Collagen homeostasis is maintained by a balance between matrix metalloproteinases (MMPs) and tissue inhibitors of metalloproteinases (TIMPs)^31^. To evaluate the protective effect of AC-EtOH against UV-induced collagen degradation, we measured the mRNA expression levels of *MMP1*, *MMP3*, and *TIMP1* in UV-irradiated WS1 cells using qPCR. As shown in Fig. 4A, UV exposure significantly upregulated the expression of *MMP1* and *MMP3*, whereas AC-EtOH treatment effectively suppressed their overexpression. Conversely, UV irradiation reduced *TIMP1* mRNA levels, but AC-EtOH treatment restored its expression. Consistently, Western blot analysis confirmed that AC-EtOH attenuated the UV-induced elevation of MMP1 and MMP3 protein levels while reversing the UV-mediated reduction of TIMP1 (Fig. 4B). These findings suggest that AC-EtOH protects against UV-induced collagen degradation. UV-induced ROS accumulation is also known to trigger the release of pro-inflammatory cytokines such as IL-6, IL-1β, and TNF-α. qPCR analysis revealed that UV irradiation significantly increased the expression of *IL-6* and *IL-1β* mRNA, and ELISA further demonstrated enhanced secretion of these cytokines following UV exposure. In contrast, AC-EtOH effectively suppressed both the transcriptional upregulation and protein secretion of these genes (Fig. 4C and D). Because NF-κB is a central mediator of inflammatory signaling in response to external stressors, including UV radiation, we next evaluated its activation status. Immunofluorescence analysis showed that UV irradiation promoted the nuclear translocation of NF-κB p65, whereas AC-EtOH reduced this UV-induced nuclear accumulation (Fig. 4E, F). Furthermore, an NF-κB luciferase reporter assay demonstrated that UV exposure significantly activated NF-κB transcriptional activity, and this activation was inhibited by AC-EtOH treatment (Fig. 4G). Together, these findings suggest that AC-EtOH mitigates UV-induced inflammation by inhibiting the activation of the NF-κB signaling pathway.

### Application and safety assessment of *A. cinnamomea* extract for skincare

**Fig. 5:**
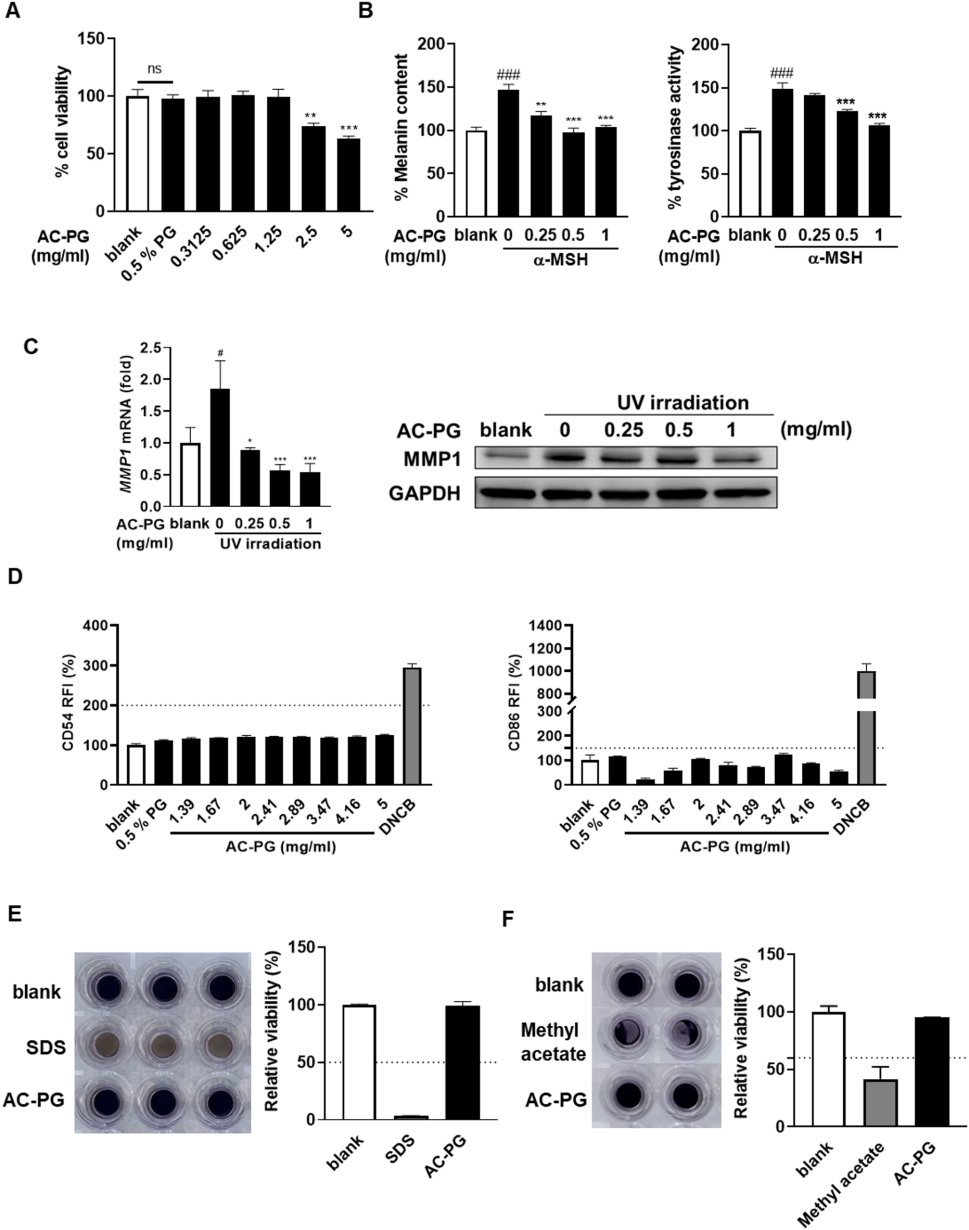
Effects of the *A. cinnamomea* extract–propylene glycol mixture (AC-PG) on melanogenesis in melanocytes and its in vitro safety evaluation. (**A**) CCK-8 assay measuring the viability of B16F10 cells treated with the indicated concentrations of AC-PG for 48 h. ###*p* < 0.001 compared to the vehicle control group. (**B**) Intracellular melanin content (left) and tyrosinase activity (right) in α-MSH–induced B16F10 cells treated with the indicated concentrations of AC-PG for 48 h. ###*p* < 0.001 compared to the vehicle control group; **p < 0.01, ***p < 0.001 compared to the α-MSH control group. (**C**) qRT-PCR (left) and Western blot (right) analyses of MMP1 expression in UV-irradiated WS1 cells treated with AC-PG for 24 h. #*p* < 0.05 compared to the non-irradiated control group; *p < 0.05 and ***p < 0.001 compared to the UV-irradiated control group. (**D**) h-CLAT assay assessing CD54 (left) and CD86 (right) expression in THP-1 cells treated with the indicated concentrations of AC-PG for 48 h. DNCB (2 µg/mL) was used as a positive control. (**E**) Representative images (left) and viability quantification (right) of reconstructed human epidermis (EpiDerm™) tissues treated with or without 2.5 mg/mL AC-PG for 24 h. 5% SDS served as a positive control for irritation. (**F**) Representative images (left) and viability quantification (right) of reconstructed human cornea-like epithelium (EpiOcular™) tissues treated with or without 2.5 mg/mL AC-PG for 2 h. Methyl acetate (50 µL) served as a positive control.

Skincare agents and cosmetics are typically formulated as hydrophilic solutions; however, AC-EtOH contains various hydrophobic bioactive compounds, including triterpenes, succinic and maleic acid derivatives, and Antcin K, making it difficult to dissolve in hydrophilic reagents^23^. Polyethylene glycol (PG) is a widely used synthetic liquid in cosmetics and skincare products that functions as a solvent for hydrophobic materials and is miscible with water^32^. Therefore, we utilized PG as a carrier solvent of AC-EtOH to develop a skincare formulation called *A. cinnamomea* extract-polyethylene glycol mixture (AC-PG), which exhibited high dispersion in PG (data not shown). A CCK-8 assay showed that AC-PG at concentrations below 1.25 mg/mL did not exert cytotoxic effects on B16F10 cells (Fig. 5A). Given the anti-melanogenic and anti-photoaging effects of AC-EtOH, we treated α-MSH-induced B16F10 and UV-irradiated WS1 cells with 0.25, 0.5, and 1 mg/mL AC-PG, corresponding to 2.5, 5, and 10 µg/mL AC-EtOH, respectively. As shown in Fig. 5B, C, AC-PG significantly reduced α-MSH–induced melanin production and tyrosinase activity, and attenuated UV-induced MMP1 expression in a dose-dependent manner, supporting its potential utility as a skin-brightening and anti-aging agent.

Skin sensitization is a common adverse effect of cosmetics and skincare products. According to the Adverse Outcome Pathway (AOP) for skin sensitization, the third key event is the activation of dendritic cells, which is marked by increased *IL-8* expression^27,33^. To assess the skin sensitization potential of AC-EtOH, we measured *IL-8* mRNA levels in THP-1 cells, a pro-dendritic cell line, and compared them to levels induced by 2,4-dinitrochlorobenzene (DNCB), a known inducer of atopic dermatitis. Cell viability assays indicated that AC-EtOH concentrations below 50 µg/mL did not affect THP-1 cell survival (Figure S2A). Thus, we performed a two-fold serial dilution starting from 50 µg/mL AC-EtOH. As shown in Figure S2B, AC-EtOH at concentrations of 25 µg/mL and 50 µg/mL significantly increased *IL-8* mRNA expression by 1.59-fold and 1.56-fold, respectively. However, compared to DNCB treatment, *IL-8* induction in the AC-EtOH-treated group was considerably lower (Figure S2B). Regarding AC-PG, concentrations above 5 mg/mL caused 45.74% cell death, whereas concentrations below 2.5 mg/mL had no significant effect on cell viability (Figure S2C). Similar to AC-EtOH, AC-PG treatment at concentrations of 2.5 mg/mL and 5 mg/mL increased *IL-8* mRNA expression by 1.45-fold and 1.50-fold (Figures S2D). To further assess sensitization potential, the Human Cell Line Activation Test (h-CLAT) was performed. 7-AAD staining confirmed that cell viability remained above 85% at AC-PG concentrations below 5 mg/mL (Figure S2E). Thus, AC-PG was subjected to 1.2-fold serial dilutions beginning at 5 mg/mL. As shown in Fig. 5D, the RFIs of CD54 and CD86 in AC-PG–treated cells did not exceed the OECD TG 442E positivity thresholds, indicating that AC-PG does not induce dendritic cell activation under the tested conditions.

Irritation potential is an additional safety consideration for cosmetic use. To evaluate skin and eye irritation, reconstructed human epidermis (RhE) and reconstructed human cornea-like epithelium (RhCE) tissues were treated with AC-PG, and viability was assessed using the MTT assay. As shown in Fig. 5E, RhE tissues retained 99.6% viability following treatment with 2.5 mg/mL AC-PG, classifying the formulation as non-irritant according to OECD TG 439. Similarly, RhCE tissues maintained 95.3% viability after exposure to 2.5 mg/mL AC-PG (Fig. 5F), indicating that AC-PG is non-irritating to the eye and is not expected to cause severe ocular damage, as per OECD TG 492.

## Discussion

Tyrosinase is a rate-limiting enzyme in melanin biosynthesis^3^. Consequently, many skin-lightening agents aim to inhibit melanin production by suppressing tyrosinase expression or activity^20^. In this study, AC-EtOH markedly reduced the expression of tyrosinase and tyrosinase-related proteins, as demonstrated by qPCR and Western blot analyses. Notably, AC-EtOH did not inhibit tyrosinase activity in a cell-free enzymatic assay, indicating that its anti-melanogenic effect is attributable to reduced tyrosinase expression rather than direct enzymatic inhibition. This mechanism differs fundamentally from that of kojic acid (KA), which suppresses melanogenesis by chelating copper ions essential for tyrosinase activity^34^. Thus, AC-EtOH and KA reduce melanogenesis through fundamentally different mechanisms. Moreover, AC-EtOH significantly downregulated MITF expression, decreased intracellular cAMP accumulation, and inhibited CREB phosphorylation. Given that MITF is a master transcription factor regulated by the PKA/cAMP/CREB pathway^7,8^, our findings suggest that AC-EtOH suppresses tyrosinase synthesis by attenuating this signaling axis. Although the upstream molecular targets of AC-EtOH within the PKA/cAMP/CREB pathway remain unclear, further biochemical and metabolomic investigations will be valuable for identifying the bioactive constituents responsible for this effect.

UVB-induced reactive oxygen species (ROS) play a key role in photoaging, triggering the release of senescence-associated secretory phenotype (SASP) factors from aging cells, including matrix metalloproteinases (MMPs) and pro-inflammatory cytokines^30^. MMPs, such as MMP1, MMP3, and MMP9, degrade collagen, contributing to wrinkle formation^35^. Pro-inflammatory cytokines, such as IL-6 and IL-8, recruit dendritic cells, neutrophils, and macrophages, thereby altering the skin microenvironment and promoting chronic inflammation^36^. It is well established that the NF-κB pathway regulates the production of pro-inflammatory cytokines^37^. UV-induced ROS activates NF-κB translocation, inducing the transcription of *IL-1β*, *IL-6*, *IL-8*, and *MMPs*^38,39^. In our study, AC-EtOH significantly reduced UV-induced ROS accumulation in skin fibroblasts and effectively counteracted UV-induced dysregulation of *MMP1*, *MMP3*, and *TIMP1* at both the mRNA and protein levels. This coordinated suppression of MMPs, coupled with the restoration of TIMP1 expression, highlights the protective role of AC-EtOH in maintaining extracellular matrix homeostasis. Additionally, AC-EtOH markedly attenuated UV-induced IL-1β and IL-6 expression and secretion. Immunofluorescence and NF-κB reporter assays further demonstrated that AC-EtOH suppresses UV-induced NF-κB activation and nuclear translocation. These observations suggest that AC-EtOH exhibits potent antioxidant and anti-inflammatory properties, mitigating the ROS-mediated upregulation of MMPs and cytokines while inhibiting NF-κB signaling. These findings align with previous reports, which show that *A. cinnamomea* extracts and their constituents possess antioxidant and cytoprotective properties across multiple tissues, including the liver, skin, and kidneys^40–42^. Antrodin C—a maleic/succinic acid derivative isolated from *A. cinnamomea mycelium*—has been shown to inhibit hyperglycemia-induced ROS, reduce cellular senescence and apoptosis, and suppress nitric oxide production in activated macrophages and microglia^43–46^. Additionally, it inhibits nitric oxide production in LPS-induced murine macrophages and microglial cells. HPLC analysis revealed that AC-EtOH contains 15.47% Antrodin C (Figure S3), suggesting that it may be a major contributor to the observed protective effects against UV-induced oxidative stress in skin fibroblasts.

Propylene glycol (PG) is widely used as a solvent, humectant, and preservative in foods, pharmaceuticals, and cosmetics^47^ and is classified as “generally recognized as safe” (GRAS) by the U.S. Food and Drug Administration^48^. AC-EtOH dissolves well in PG, forming a homogeneous solution without aggregation or precipitation, which we have named AC-PG. In a melanin assay and tyrosinase activity assay, AC-PG significantly reduced melanin production and tyrosinase activity in α-MSH-induced B16F10 cells and suppressed UV-induced MMP1 expression in WS1-irradiated cells. Notably, the anti-melanogenic and anti-photoaging efficacy of AC-PG was comparable to that of AC-EtOH alone, indicating that PG is an effective solvent that does not diminish AC-EtOH’s biological activity.

The outermost layer of the skin, the stratum corneum (SC), serves as a primary barrier against transdermal delivery^49,50^. Studies have reported that PG interacts with intercellular lipids in the SC, decreasing barrier function and enhancing molecule permeation through the skin^51,52^. Given that the target cells of AC-EtOH—melanocytes and fibroblasts—are located in the basal epidermis and dermis^49,53^, dissolving AC-EtOH in PG may enhance its penetration, making it a promising candidate for cosmetic, skincare, and topical medicinal applications.

Skin sensitization and irritation assessment are crucial steps in the development of cosmetics and skincare products. Traditionally, this assessment relied on animal testing, such as *the in vivo Draize eye test*,* skin irritation test*, and the mouse local lymph node assay (LLNA). With the growing trend toward replacing animal testing, alternative methods have been developed. Based on the key events of the Adverse Outcome Pathway (AOP) framework for skin sensitization, Dendritic cell (DC) activation represents the third key event in this pathway^33^. OECD TG 442E recommends assays such as the THP-1 IL-8 reporter gene assay and the human cell line activation test (h-CLAT) for identifying potential sensitizers^27^. A positive response is defined as an IL-8 luciferase activity level more than 1.4-fold higher than the control^33^. In our study, AC-EtOH concentrations above 25 µg/mL (and AC-PG above 2.5 mg/mL, corresponding to equivalent AC-EtOH content) induced *IL-8* upregulation in THP-1 cells. However, the h-CLAT results showed that even the highest AC-PG concentration tested (5 mg/mL) failed to exceed the CD86 and CD54 positivity thresholds (150% and 200%, respectively), indicating a negative sensitization response. Overall, these results suggest that AC-PG concentrations above 2.5 mg/mL may carry a sensitization risk, while lower concentrations remain within safe ranges.

Irritation potential was assessed using reconstructed human epidermis (RhE) and cornea-like epithelium (RhCE) tissue models. AC-PG at 2.5 mg/mL did not exhibit irritant effects in either model, corresponding to UN GHS “No Category” classification. Given that the effective concentrations of AC-PG for anti-melanogenic and anti-photoaging effects are below 1 mg/mL, we recommend that topical products utilize lower doses to minimize the risk of sensitization and irritation while maintaining biological efficacy.

**Fig. 6:**
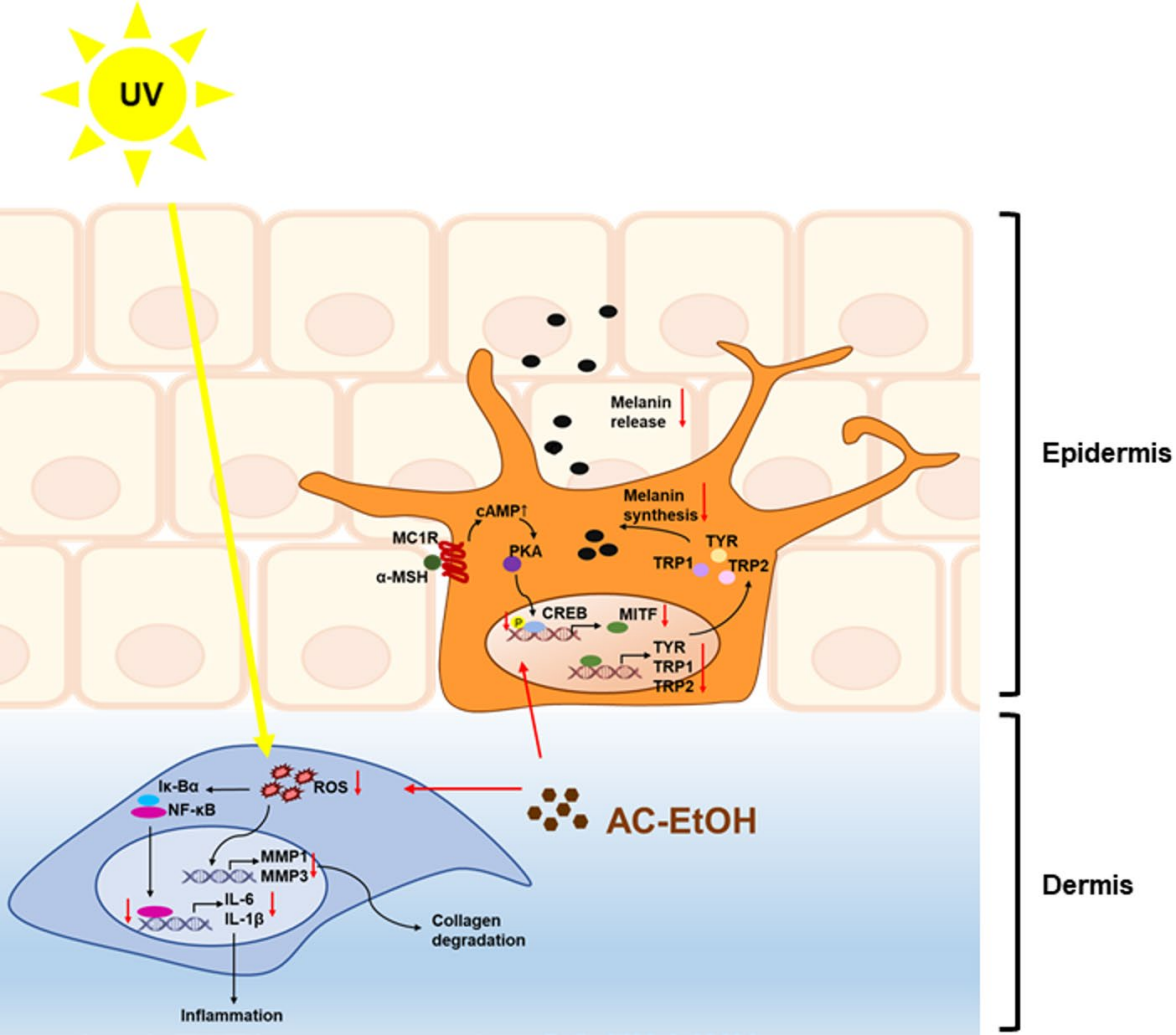
Model of *A. cinnamomea* extract mediated anti-melanogenesis and anti-photoaging effects. *A. cinnamomea* extract inhibits melanin production by reducing tyrosinase synthesis through the blockade of the PKA/cAMP/CREB pathway. Additionally, *A. cinnamomea* extract reduces ROS accumulation, thereby ameliorating ROS-induced inflammation and collagen degradation, which contributes to its anti-photoaging effects.

In conclusion, AC-EtOH significantly inhibits α-MSH–induced melanogenesis by downregulating tyrosinase and related enzymes, primarily through suppression of MITF expression via the PKA/cAMP/CREB pathway. Additionally, AC-EtOH protects skin fibroblasts from UV-induced collagen degradation, oxidative stress, and inflammation (Fig. 6). Together with its favorable safety profile and lack of irritation at effective concentrations, AC-EtOH represents a promising candidate for the development of cosmetic and skincare formulations.

## Supplementary Information

Below is the link to the electronic supplementary material.

Supplementary Material 1

Supplementary Material 2

## Data Availability

All data supporting the findings of this study are available within the paper and its Supplementary Information. Primer and probe sequences are provided in Supplementary Table S1.
