# Supplementary material for: Antrodia cinnamomea extract exhibits anti-melanogenic and anti-photoaging effects: potential for cosmetic and dermatological applications
Source: Sci Rep. 2026 May 28;16:24448. doi: 10.1038/s41598-026-54214-8 (PMC13448119; doi:10.1038/s41598-026-54214-8)
Supplement: Supplementary file 1 [file 41598_2026_54214_MOESM1_ESM.docx]

***Antrodia cinnamomea* extract exhibits anti-melanogenic and anti-photoaging effects: potential for cosmetic and dermatological applications**

I-Hsuan Huang^1,2^, Tse-Kai Fu^2^, Yu-Ting Chou^1*^

^1^Institute of Biotechnology, National Tsing Hua University, Hsinchu, Taiwan.

^2^Simpson Biotech Co., Ltd., Taoyuan, Taiwan.

*Corresponding author

Yu-Ting Chou, National Tsing Hua University, No. 101, Section 2, Kuang-Fu Road, Hsinchu 30013, Taiwan. Phone: 886-3574-2471; E-mail: ytchou@life.nthu.edu.tw

**Figure S1. *A. cinnamomea extract reduces UV-induced ROS in WS1 skin fibroblasts*.**

(A) qRT-PCR analysis of *IL-1β* mRNA expression in WS1 cells exposed to the indicated UV dosages. *** *p <* 0.001 compared to the non-irradiated control group.

(B) Representative images (left) of DCF-DA staining in WS1 cells treated with or without 40 mJ/cm² UVB and then treated with the indicated concentrations of AC-EtOH for 24 h. Quantification (right) of intracellular ROS levels based on DCF-DA fluorescence intensity. N-acetylcysteine (NAC; 1 mM) was used as a positive control. Scale bars, 200 μm. ### *p <* 0.001 compared to the non-irradiated control group; *** *p <* 0.001 compared to the UV-irradiated control group.

**Figure S2. Effects of the *A. cinnamomea* extract–propylene glycol mixture (AC-PG) on cell viability and IL-8 induction in pro-dendritic THP-1 cells.**

(A) CCK-8 assay measuring the viability of THP-1 cells treated with the indicated concentrations of AC-EtOH for 48 h. ## *p <* 0.01, ### *p <* 0.001 compared to the vehicle control group.

(B) qRT-PCR analysis of *IL-8* mRNA expression in THP-1 cells treated with AC-EtOH for 24 h. ## *p <* 0.01, ### *p <* 0.001 compared to the vehicle control group.

(C) CCK-8 assay measuring the viability of THP-1 cells treated with the indicated concentrations of AC-PG for 48 h. ### *p <* 0.001 compared to the vehicle control group.

(D) qRT-PCR analysis of *IL-8* mRNA expression in THP-1 cells treated with AC-PG for 24 h. ## *p <* 0.01, ### *p <* 0.001 compared to the vehicle control group.

(E) Flow cytometry analysis showing cell viability (7-AAD staining) of THP-1 cells treated with AC-PG for 24 h, used to determine non-cytotoxic concentrations for the h-CLAT assay.

**Figure S3. HPLC chromatogram of AC-EtOH.**

High-performance liquid chromatography (HPLC) chromatogram of AC-EtOH showing the presence and relative abundance of major constituents, including Antrodin C.

**Table S1. Primer and probe list**

| **UPL primer sequence** | |  |  |
| --- | --- | --- | --- |
| Name | Sequence | UPL Probe | Accession No |
| *Gapdh* F | GTCAAGGCCGAGAATGGGAA | #9 | NM_001289726.2 |
| *Gapdh* R | TGATGTTAGTGGGGTCTCGC |  |  |
| *Mitf* F | CGACAGAAGAAGCTGGAGCA | #62 | NM_001113198.2 |
| *Mitf* R | CTAGCCTGCATCTCCAGCTC |  |  |
| *Tyr* F | TCGTATCTGGCCATGGCTTC | #24 | NM_001317397.2 |
| *Tyr* R | CTAAGCCCAGAGAGAGCTGC |  |  |
| *Tyrp-1* F | GACTGACCCTTGTGGCTCAT | #34 | NM_001282014.1 |
| *Tyrp-1* R | GTCGGGAGTCTGCAATCACA |  |  |
| *Tyrp-2* F | AGACAGGTCTCAGCAGCAAG | #69 | NM_010024.3 |
| *Tyrp-2* R | TTACTACCCAGGTCAGGCCA |  |  |
| *MMP1* F | AGGGGAGATCATCGGGACAA | #85 | NM_001145938.2 |
| *MMP1* R | ACCTGGGCCTGGTTGAAAAG |  |  |
| *MMP3* F | ATCCTACTGTTGCTGTGCGT | #58 | NM_002422.5 |
| *MMP3* R | GGTTCATGCTGGTGTCCTCA |  |  |
| *TIMP1 F* | CAAACTGCAGAGTGGCACTC | #17 | NM_003254.3 |
| *TIMP1 R* | GGGACTGGAAGCCCTTTTCA |  |  |
| *IL-6* F | TTCGGTCCAGTTGCCTTCTC | #40 | NM_000600.5 |
| *IL-6* R | TCTTCTCCTGGGGGTACTGG |  |  |
| **Taqman primer sequence** | |  |  |
| Name | Sequence |  | Accession No |
| *18S F* | TGGCTCATTAAATCAGTTATG |  |  |
| *18S R* | CGGCATGTATTAGCTCTA |  |  |
| *IL-1 β* F | ACCTCCAGGGACAGGATATGG |  |  |
| *IL-1 β* R | CCCAAGGCCACAGGTATTTTG |  |  |
| **Taqman probe sequence** | |  |  |
| Name | Sequence |  |  |
| *18S* | 56-FAM/CGCTCGCTC/ZEN/CTCTCCTACTTG/3IABkFQ | | |
| *IL-1 β* | 56-FAM/AACAAGTGG/ZEN/TGTTCTC/3IABkFQ | | |
